# Supplementary material for: The prevalence of suicidal behavior and its associated factors among wives with polygamy marriage living in Gedeo zone, southern Ethiopia, 2020
Source: PLoS One. 2021 Oct 25;16(10):e0259029. doi: 10.1371/journal.pone.0259029 (PMC8544854; doi:10.1371/journal.pone.0259029)
Supplement: Attachment — Submitted filename: PONE-D-21-07591_reviewer#1.pdf [file pone.0259029.s001.pdf]

PLOS ONE

The prevalence of suicidal behavior and its associated factors among wives with polygamy marriage living in Gedeo zone, Southern Ethiopia, 2020.

--Manuscript Draft--

Manuscript Number:	PONE-D-21-07591
Article Type:	Research Article
Full Title:	The prevalence of suicidal behavior and its associated factors among wives with polygamy marriage living in Gedeo zone, Southern Ethiopia, 2020.
Short Title:	Suicidal behavior among wives with polygamy marriage
Corresponding Author:	Seid Shumye<br>Dilla University College of Health Sciences<br>Addis Ababa, ETHIOPIA
Keywords:	Keyword: Polygamy; Women; Suicidal behavior; Ethiopia
Abstract:	Abstract<br>Introduction : Polygamy is a trend of marriage characterized by having two and more wives or husbands at the same time. In low and middle-income countries like Ethiopia, polygamy has a significant negative effect on the social, economical, physical, and mental well-being of women. Therefore, this study aimed to assess the magnitude and associated factors of suicidal behavior among wives with polygamy marriage living in the Gedeo zone, Southern Ethiopia, 2020.<br>Methods : A community-based cross-sectional study design and systematic sampling technique was employed among wives with polygamy marriage who are living in Gedeo zone from Nov – Dec 2020. Suicidal behavior revised questioner (SBQ-5) were used to assess the outcome variable. The Logistic regression at 95% CI, p<0.05 was used to identify factors associated with suicidal behavior.<br>Results: This study enrolled 423 respondents. The study revealed that, the overall prevalence of suicidal behavior was 157(37%). Illiteracy, being a wife of a husband with three and more other wives, current history of depression, intimate partner violence, and poor social support were significantly associated with suicidal behavior at 95% confidence interval, p < 0.05.<br>Conclusion: This study found that one-third of the respondents had suicidal behavior, and Illiteracy, being a wife of a husband with three and more other wives, poor social support, current history of depression, and intimate partner violence were the most affected group of respondents. Therefore, due attention should be given to minimize the practice and its effect on the mental wellbeing of a mother and their children.
Order of Authors:	Seid Shumye
	Chalachew Kassaw
Additional Information:	
Question	Response
**Financial Disclosure**<br><br>Enter a financial disclosure statement that describes the sources of funding for the work included in this submission. Review the submission guidelines for detailed requirements. View published research articles from *PLOS ONE* for specific examples.<br><br>This statement is required for submission and **will appear in the published article** if the submission is accepted. Please make	The author(s) received no specific funding for this work

Powered by Editorial Manager® and ProduXion Manager® from Aries Systems Corporation

sure it is accurate.<br><br>**Unfunded studies**<br>Enter: *The author(s) received no specific funding for this work.*<br><br>**Funded studies**<br>Enter a statement with the following details:<br>• Initials of the authors who received each award<br>• Grant numbers awarded to each author<br>• The full name of each funder<br>• URL of each funder website<br>• Did the sponsors or funders play any role in the study design, data collection and analysis, decision to publish, or preparation of the manuscript?<br>• **NO** - Include this sentence at the end of your statement: *The funders had no role in study design, data collection and analysis, decision to publish, or preparation of the manuscript.*<br>• **YES** - Specify the role(s) played.<br><br>* typeset	
**Competing Interests**<br><br>Use the instructions below to enter a competing interest statement for this submission. On behalf of all authors, disclose any competing interests that could be perceived to bias this work—acknowledging all financial support and any other relevant financial or non-financial competing interests.<br><br>This statement is required for submission and **will appear in the published article** if the submission is accepted. Please make sure it is accurate and that any funding sources listed in your Funding Information later in the submission form are also declared in your Financial Disclosure statement.<br><br>View published research articles from *PLOS ONE* for specific examples.	The authors have declared that no competing interests exist.

**NO authors have competing interests**<br><br>Enter: *The authors have declared that no competing interests exist.*<br><br>**Authors with competing interests**<br><br>Enter competing interest details beginning with this statement:<br><br>*I have read the journal's policy and the authors of this manuscript have the following competing interests: [insert competing interests here]*<br><br>* typeset	
**Ethics Statement**<br><br>Enter an ethics statement for this submission. This statement is required if the study involved:<br><br>• Human participants<br>• Human specimens or tissue<br>• Vertebrate animals or cephalopods<br>• Vertebrate embryos or tissues<br>• Field research<br><br>Write "N/A" if the submission does not require an ethics statement.<br><br>General guidance is provided below. Consult the submission guidelines for detailed instructions. **Make sure that all information entered here is included in the Methods section of the manuscript.**	Ethical clearance was obtained from the ethical review board of Gedeo zonal women and child health beauro. Each participant has given written informed consent to participate in the current study. The study ensured issues of voluntary participation and Confidentiality throughout the data collection period.

**Format for specific study types**<br><br>**Human Subject Research (involving human participants and/or tissue)**<br>• Give the name of the institutional review board or ethics committee that approved the study<br>• Include the approval number and/or a statement indicating approval of this research<br>• Indicate the form of consent obtained (written/oral) or the reason that consent was not obtained (e.g. the data were analyzed anonymously)<br><br>**Animal Research (involving vertebrate animals, embryos or tissues)**<br>• Provide the name of the Institutional Animal Care and Use Committee (IACUC) or other relevant ethics board that reviewed the study protocol, and indicate whether they approved this research or granted a formal waiver of ethical approval<br>• Include an approval number if one was obtained<br>• If the study involved *non-human primates*, add *additional details* about animal welfare and steps taken to ameliorate suffering<br>• If anesthesia, euthanasia, or any kind of animal sacrifice is part of the study, include briefly which substances and/or methods were applied<br><br>**Field Research**<br><br>Include the following details if this study involves the collection of plant, animal, or other materials from a natural setting:<br>• Field permit number<br>• Name of the institution or relevant body that granted permission	
**Data Availability**<br><br>Authors are required to make all data underlying the findings described fully available, without restriction, and from the time of publication. PLOS allows rare exceptions to address legal and ethical concerns. See the PLOS Data Policy and FAQ for detailed information.	Yes - all data are fully available without restriction

Powered by Editorial Manager® and ProduXion Manager® from Aries Systems Corporation

A Data Availability Statement describing where the data can be found is required at submission. Your answers to this question constitute the Data Availability Statement and **will be published in the article**, if accepted.<br><br>**Important:** Stating 'data available on request from the author' is not sufficient. If your data are only available upon request, select 'No' for the first question and explain your exceptional situation in the text box.<br><br>Do the authors confirm that all data underlying the findings described in their manuscript are fully available without restriction?	
**Describe where the data may be found in full sentences. If you are copying our sample text, replace any instances of XXX with the appropriate details.**<br><br>• If the data are **held or will be held in a public repository**, include URLs, accession numbers or DOIs. If this information will only be available after acceptance, indicate this by ticking the box below. For example: *All XXX files are available from the XXX database (accession number(s) XXX, XXX.*).<br>• If the data are all contained **within the manuscript and/or Supporting Information files**, enter the following: *All relevant data are within the manuscript and its Supporting Information files.*<br>• If neither of these applies but you are able to provide **details of access elsewhere**, with or without limitations, please do so. For example:<br><br>*Data cannot be shared publicly because of [XXX]. Data are available from the XXX Institutional Data Access / Ethics Committee (contact via XXX) for researchers who meet the criteria for access to confidential data.*<br><br>*The data underlying the results presented in the study are available from (include the name of the third party*	The data used for the study was available on the Corresponding Email author based on a reasonable request.

and contact information or URL).<br>• This text is appropriate if the data are owned by a third party and authors do not have permission to share the data.<br><br>* typeset	
Additional data availability information:	

manuscript Click here to access/download;Manuscript;new manscript clean polygamy.docx

The prevalence of suicidal behavior and its associated factors among wives with polygamy marriage living in Gedeo zone, Southern Ethiopia, 2020.

Chalachew Kassaw [1] Seid Shumye *[2]

1Department of Psychiatry, College of Health Science, Dilla University, P.O. Box 419) email, (1234berekassa@gmail.com), Dilla University, Dilla, Ethiopia.

2[*] Department of Psychiatry, College of Health Science, Dilla University, P.O. Box 419) email, (seidshumye22@gmail.com), Dilla University, Dilla, Ethiopia.

Corresponding Author: Seid Shumye, Department of Psychiatry, College of Health Science, Dilla University, and Dilla, Ethiopia.

Email: (seidshumye22@gmail.com)

Mobile Phone number: +251926023903

Abstract

Introduction: Polygamy is a trend of marriage characterized by having two and more wives or husbands at the same time. In low and middle-income countries like Ethiopia, polygamy has a significant negative effect on the social, economical, physical, and mental well-being of women. Therefore, this study aimed to assess the magnitude and associated factors of suicidal behavior among wives with polygamy marriage living in the Gedeo zone, Southern Ethiopia, 2020.

Methods: A community-based cross-sectional study design and systematic sampling technique was employed among wives with polygamy marriage who are living in Gedeo zone from Nov – Dec 2020. Suicidal behavior revised questioner (SBQ-5) were used to assess the outcome variable. The Logistic regression at 95% CI, $p<0.05$ was used to identify factors associated with suicidal behavior.

Results: This study enrolled 423 respondents. The study revealed that, the overall prevalence of suicidal behavior was 157(37%). Illiteracy, being a wife of a husband with three and more other wives, current history of depression, intimate partner violence, and poor social support were significantly associated with suicidal behavior at 95% confidence interval, $p < 0.05$.

Conclusion: This study found that one-third of the respondents had suicidal behavior, and Illiteracy, being a wife of a husband with three and more other wives, poor social support, current history of depression, and intimate partner violence were the most affected group of respondents. Therefore, due attention should be given to minimize the practice and its effect on the mental wellbeing of a mother and their children.

Keyword: Polygamy; Women; Suicidal behavior; Ethiopia

Introduction

Suicidal behavior is a pattern of thinking or predispositions that situated an individual at risk of committing suicide. Suicidal behavior has three categories; suicide ideation, suicide plan or intent, and suicide attempts which are the leading cause of harm and end of life(1). Suicide is one of the complications of a psychiatric disorder to affect all individuals irrespective of their nation, culture, religion, gender, and class. Currently, Lithuanians rank first for a suicidal rate, which accounts 31/100k(2). Globally, while over 16,000,000 people attempt suicide, and 800,000 commit a suicide every year, other 3000 individuals also commit a suicide in a daily base. The case is more prevalent among youths and women (3).

Polygamy is a state of marriage in which a husband marries more than two wives at a time and remains one of the hotly contested and acceptable practices. It is a misunderstood topic in the traditional and modern society of Africa and Ethiopia. It is acceptable in most cultures and religions, particularly in Muslim (4-6). It is a very problematic and well-buried practice against freedom, equality, and human rights of a woman(7). It has also a burden on husbands to care for all their wives and children in terms of social, economic, psychological, and physical needs(8).

Psychological and social factors such as jealousy, intimate partner violence, lack of social support, and economic dependency on their husband were contributing factors for having mental disorders among women with polygamy marriage (9-11). A woman with polygamy marriage was reported repeated physical and psychological violence from their husband; Children showed feelings of pain, hurt, and anger. Moreover, in low and middle-level countries, it has a risk for having sexually transmitted disease, including HIV/AIDS which also contribute for mental health problems on themselves, family and society at all(12, 13). Psychiatric problems such as mood, anxiety, and psychosis were documented among woman with polygamy marriage in particular among senior wives and their family members(14, 15). Developed countries were launched human and legal rights to prevent any forms of women abuse and violence happened during marriage(16). This resulted in reduction of polygamy marriage in western countries. However, in Ethiopia, polygamy cultural practice is still highly prevalent and its effect on the mental and psychological health of wives is not well studied(7). Therefore, this study aimed to assess the magnitude and associated factor of suicidal behavior among wives with polygamy marriage living in Gedeo zone, southern Ethiopia, 2021.

Methods

Study Design, Setting and Period

This study was a community-based cross-sectional study design conducted in Gedeo zone among wives with polygamy marriage from Nov – Dec 2020. A systematic random sampling technique was used to collect the required number of samples from the study population. Polygamy marriage is highly practiced and accepted to the extent of marrying four wives at a time in the current study area, the Gedeo zone(8). The zone has a population of 850, 534 peoples and 500,000 of them were females. The fertility rate is high in this area as compared to other parts of the country. It is 359 KM far from the capital city of Ethiopia, Addis Ababa.

Study Participants

Age, which is above 18 years old and a current history of polygamy marriage have been used as a parameter to select participants for the study. Respondents with acute or severe illness and who were not at home during the data collection period were excluded from the study.

Sample Size and Sampling Technique

This study enrolled 423 respondents selected using a systematic random sampling technique.

Data Collection and Instruments

The data was collected by ten health extension workers who are working in the zonal health office by using a structured interview technique. The first part of the questioner was about the socio-demographic characteristics of the respondents. The second part of the questioner was the suicidal behavioral revised questioner (SBQ-R) used to assess suicidal behavior of the respondents related to polygamy marriage, and a score ≥ of 7/18 considered as suicidal behavior(17). The tool has 93% sensitivity and 95% specificity. The third part of a questioner was intimate partner violence used to measure intimate partner violence and score > 10/25 classified as intimate partner violence(18). The fourth part of the questioner was the PHQ-9 tool used to assess depression, and a score > 10/21 said to have depression(19). The final part of the questioner was the Oslo social support scale (Oslo -3) used to measure social support levels ranging from 3 to 14 and classified as poor (3-8), moderate (9-11), and strong social support (12-14)(20).

Data Quality Control

To assure the data quality and consistency, the English version of the questioner was translated to Amharic and Gedeoffa, the local language of the study area then, back-translated to English by a language expert. The study used a Gedeoffa version questioner for the data collection and conducted a pretest before two weeks of the concrete data collection period on the 20 (5%) respondents. Both data collectors and investigators checked data for its completeness daily.

Data Management and Analysis

Data was entered into the Epi-Data version 3.4 software package and exported to the Statistical Package for Social Science version 22. Descriptive statistics; frequency, percentage, mean and standard deviation were used to describe the socio-demographic characteristics of respondents. A logistic regression analysis at $p \leq 0.05$, 95% CI was used to interpret the association with the independent variables.

Result

Socio-demographic results of respondents

The mean (SD) age of respondents was 30 (±6) years old, more than half of them were living in rural areas, and more than two-thirds of the respondents have no formal education (Table 1.)

Table1.Socio-demographic respondents at Gedeo zone, Southern Ethiopia, Dec, 2020 (N=423).

variables	Category	Frequency	Percentage
Age	Below 30	135	32 %
	Above 30	288	68%
Educational status	Non formal(Illiterate)	309	73%
	Primary school	114	27%
Residence	Urban	42	10%
	Rural	381	90%
Husbands work	Farmer	313	74%
	Merchant	110	26%
Wives work	House wife	250	59%
	Farmer	89	21%
	Merchant	84	20%
Religion	Protestant	329	77.8
	Orthodox	64	15.1
	Muslim	30	7.1

Number of wives at a time	Two	300	71%
	Three	123	29%
Distance of the two wives house	< 20 km	148	35%
	>20 km	275	65%
Family size	<3	157	37%
	>3	266	63%
Social support	Poor	182	43%
	Moderate	89	21%
	Strong	152	36%
Duration of marriage	< 3 year	63	15%
	3-6 year	148	35%
	>6year	212	50%

Psychosocial results of respondents

According to this study result, 237 (56%) of respondents had depression, 186 (44%) intimate partner violence, and 157 (37%) suicidal behavior.

Factors associated with suicidal behavior

During multivariable logistic regression analysis, at 95% CI ($p < 0.05$), the independent variables such as educational status, number of wives, history of depression, intimate partner violence, and poor social support were associated with the outcome variable (Table 2).

Table .2.Variables associated with suicidal behavior, Southern Ethiopia, Dec, 2020 (N=423).

Variables	Category variables		Suicidal behavior		Adjusted odds ratio (AOR)	P-value
			Yes	No		
Age	Below 30	135	94	41	1.48(0.96, 2.29)	
	Above 30	288	175	113	1	
Educational status	Non formal(illiterate)	309	211	98	3.83 (2.44-6.02)	0.001*
	Primary school	114	41	73	1	
Residence	Urban	42	19	23	0.53(0.28,1.01)	
	Rural	381	232	149	1	
Husbands work	Farmer	313	140	173	1	
	Merchant	110	43	67	0.79(0.51,1.24)	

Wives work	House wife	250	134	116	1	
	Farmer	89	39	50	0.68(0.41,1.10)	
	Merchant	84	52	32	1.41(0.85, 2.33)	
Number of wives at a time	Two	300	132	168	1	
	Three	123	79	44	2.29(1.48,3.53)	0.01*
Distance of the two wives house	< 20 km	148	94	54	1.21(0.80,1.83)	
	>20 km	275	162	113	1	
Family size	<3	157	95	62	1.17(0.78,1.74)	
	>3	266	151	115	1	
Depression	Yes	237	132	105	2.37(1.60-3.52)	0.001**
	No	186	65	120	1	
Intimate partner violence	Yes	144	90	54	3.18(2.09,4.83)	0.02*
	No	279	96	183	1	
Social support	Poor	182	106	76	3.02(1.92, 4.75)	0.01*
	Moderate	89	36	53	1.47 (0.85,2.54)	
	Strong	152	48	104	1	
Duration of marriage	< 3 year	63	38	25	0.94(0.53, 1.67)	
	3-6 year	148	99	49	1.25(0.80, 1.94)	
	>6year	212	131	81	1	

Discussion

This community-based cross-sectional study design aimed to assess the magnitude of suicidal behavior and its associated factors among wives with polygamy marriage living in the Gedeo zone. This study found that 157 (37%) of the respondents had suicidal behavior. The determinant factors for experiencing suicidal behavior were those with non-formal educational statuses, number of wives, current depression, intimate partner violence, and poor social support.

This study found that 157 (37 %) of the respondents had suicidal behavior, which was higher than the study conducted in Peru (22.6%)(21), Thailand (17.6%)(22), and Ethiopia (14%)(23). The possible explanation for this higher magnitude of suicidal behavior in current study could be the cultural difference and contextual definition of having more than one wife. A polygamous

marriage is related to a lack of emotional support, economic dependency, and the burden of care of children, which all be the contributing risk factors to suicidal behavior.

This study found that respondents with no formal education/ illiterate had 3.83 times (2.44-6.02) more likely to develop suicidal behavior than those with primary education, which was supported by the study conducted in the United States (24)and low and middle-level countries including, Ethiopia(25). Those with no formal education have lack of awareness, knowledge, and understanding regarding the women's rights, family, and marriage law of a country, which are vital to prevent intimate partner violence and abuse. Moreover, there is a tendency to be culturally more influenced in sharing their feelings frankly, opinions, and doubts happening in their marriage.

According to this study finding, being a wife of a husband with three and more other wives were 2.29 times (1.48, 3.53) more likely to experience suicidal behavior as compared to those with two wives which, was supported by the study done in Norway(26). As the numbers of wives increase, the challenges of polygamy marriage such as social, emotional, economic crises become significant, which are common reasons for low self-esteem, helplessness, hopelessness, and thoughts of harming self.

Respondents with a current history of depression had 2.37 times (1.60-3.52) more likely to develop suicidal behavior, which was similar to the previous study done in Germany(27).

Depression is a common psychiatric disorder characterized by loss of interest, negative attitude for self, others, and the future, which all lead to the thoughts and actions of harming self.

According to this study result, respondents with a history of intimate partner violence had 3.18 times (2.09, 4.83) more likely to experience Suicidal behavior, which was similar to the studies conducted in Thailand(22) and Korea(28). Intimate partner violence has negative health consequence on the physical, emotional, psychological, and mental health of married women, which gradually cause serious psychiatry emergency conditions such as suicidal behavior.

The last predictor variable related to suicidal behavior was women's social support, and those with poor social support had 3.02 times (1.92, 4.75) more likely to develop suicidal behavior as compared to those with strong social support which was supported by the study done in 23 European countries(29). Social support is a pillar for filling the gap of economic, social, and emotional crisis observed on women with polygamy marriage. Having no support results in

helplessness, worthlessness, and low self-esteem, which are the main entry symptoms of depression and suicidal behavior.

Conclusion

This study found that more than one-third of women with polygamy marriage had suicidal behavior, and the most affected groups were those with no formal education, triple polygamy, and current history of depression, intimate partner violence, and poor social support. Therefore, this harmful traditional and cultural practice should be avoided and reduced through the collaborative work of traditional and local community leaders, local and international women's rights association organizations, human rights associations, religious leaders, community mental health workers, and the ministry of health in the country level. Almost all peoples living in this study area are protestant religion followers, and polygamy is against Christianity or the holy bible commandment. So, it would be better if leaders of the religion teach them the right and acceptable types of marriage in Christianity, which is an essential weapon to stop this type of marriage.

List abbreviations

AOR - Adjusted odd ratio

CI- Confidence interval

OSLO -3- Oslo social support scale

PHQ- Patient health questioner

SBQ-R- Suicidal behavioral revised questioner

SD- Standard deviation

Declarations

Ethics approval and consent to participate

Ethical clearance was obtained from the ethical review board of Gedeo zonal women and child health beauro. Each participant has given written informed consent to participate in the current study. The study ensured issues of voluntary participation and confidentiality throughout the data collection period.

Consent for publication

"Not applicable".

Availability of data and materials

The data used for the study was available on the corresponding author based on a reasonable request.

Competing interests

"The author declare that there is no competing interests"

Funding

No funding was secured for this study.

Authors' contributions

The author participated in study design, data entry, analysis, and interpretation of data and gave final approval of the manuscript.

Acknowledgments

The author would like to thank study participants, Gedeo zone women counsel, and Dilla University for their invaluable support and recognition.

Reference

1. Levi-Belz Y, Gvion Y, Grisaru S, Apter A. When the pain becomes unbearable: case-control study of mental pain characteristics among medically serious suicide attempters. Archives of Suicide Research. 2018;22(3):380-93.
2. Tsypes A, Owens M, Gibb BE. Suicidal ideation and attentional biases in children: An eye-tracking study. Journal of affective disorders. 2017;222:133-7.
3. Schifano F, Napoletano F, Arillotta D, Zangani C, Gilgar L, Guirguis A, et al. The clinical challenges of synthetic cathinones. British journal of clinical pharmacology. 2020;86(3):410-9.
4. Thobejane TD, Flora T. An exploration of polygamous marriages: A worldview. Mediterranean Journal of Social Sciences. 2014;5(27 P2):1058.
5. Ugwu H. THE AFRICAN IDENTIFY OF BIBLICAL MOSES: AN IGWEBUIKE DISCOURSE. IGWEBUIKE: African Journal of Arts and Humanities. 2020;6(9).
6. Timæus IM, Reynar A. Polygynists and their wives in sub-Saharan Africa: an analysis of five Demographic and Health Surveys. Population Studies. 1998;52(2):145-62.
7. Douki S, Zineb SB, Nacef F, Halbreich U. Women's mental health in the Muslim world: Cultural, religious, and social issues. Journal of affective disorders. 2007;102(1-3):177-89.
8. Tesfay GH. Note on: the adverse effects of polygamy on the rights of women: a case study in Gedeo and Sidama Zones. Haramaya Law Review. 2017;6:91-110.
9. Dissa Y. Polygamy in Mali: social and economic implications on families. International Journal of African and Asian Studies. 2016.
10. Dervic K, Oquendo MA, Grunebaum MF, Ellis S, Burke AK, Mann JJ. Religious affiliation and suicide attempt. American Journal of Psychiatry. 2004;161(12):2303-8.
11. Barclay KJ, Thorén RD, Hanson HA, Smith KR. The Effects of Marital Status, Fertility, and Bereavement on Adult Mortality in Polygamous and Monogamous Households: Evidence From the Utah Population Database. Demography. 2020;57(6):2169-98.
12. Simkhada P, Van Teijlingen E, Winter R, Fanning C, Dhungel A, Marahatta S. Why are so many Nepali women killing themselves? A review of key issues. Journal of Manmohan Memorial Institute of Health Sciences. 2015;1(4):43-9.
13. Alhuzail NA. Being a girl in a polygamous family Implications and challenges. Child and Adolescent Social Work Journal. 2020;37(1):97-107.
14. Shepard LD. The impact of polygamy on women's mental health: a systematic review. Epidemiology and psychiatric sciences. 2013;22(1):47.
15. Devries K, Watts C, Yoshihama M, Kiss L, Schraiber LB, Deyessa N, et al. Violence against women is strongly associated with suicide attempts: evidence from the WHO multi-country study on women's health and domestic violence against women. Social science & medicine. 2011;73(1):79-86.
16. Fulu E, Warner X, Miedema S, Jewkes R, Roselli T, Lang J. Why do some men use violence against women and how can we prevent it? Quantitative findings from the United Nations multi-country study on men and violence in Asia and the Pacific. 2013.
17. Osman A, Bagge CL, Gutierrez PM, Konick LC, Kopper BA, Barrios FX. The Suicidal Behaviors Questionnaire-Revised (SBQ-R): validation with clinical and nonclinical samples. Assessment. 2001;8(4):443-54.
18. Rabin RF, Jennings JM, Campbell JC, Bair-Merritt MH. Intimate partner violence screening tools: a systematic review. American journal of preventive medicine. 2009;36(5):439-45. e4.
19. Arroll B, Goodyear-Smith F, Crengle S, Gunn J, Kerse N, Fishman T, et al. Validation of PHQ-2 and PHQ-9 to screen for major depression in the primary care population. The annals of family medicine. 2010;8(4):348-53.

20. Dalgard O. Social support-Consequences for individual and society. EUPHIX, EUphact Bilthoven: RIVM,< http://www euphix org> EUphact\Determinants of health\Environment\Social support. 2009;16.
21. Levey EJ, Rondon MB, Sanchez S, Zhong Q-Y, Williams MA, Gelaye B. Suicide risk assessment: examining transitions in suicidal behaviors among pregnant women in Perú. Archives of women's mental health. 2019;22(1):65-73.
22. Peltzer K, Pengpid S. Associations between intimate partner violence, depression, and suicidal behavior among women attending antenatal and general outpatients hospital services in Thailand. Nigerian journal of clinical practice. 2017;20(7):892-9.
23. Belete H, Misgan E. Suicidal behaviour in postnatal mothers in northwestern Ethiopia: a cross-sectional study. BMJ open. 2019;9(9):e027449.
24. Cramer RJ, Langhinrichsen-Rohling J, Kaniuka AR, Wilsey CN, Mennicke A, Wright S, et al. Preferences in information processing, marginalized identity, and non-monogamy: understanding factors in suicide-related behavior among members of the alternative sexuality community. International journal of environmental research and public health. 2020;17(9):3233.
25. Jordans M, Rathod S, Fekadu A, Medhin G, Kigozi F, Kohrt B, et al. Suicidal ideation and behaviour among community and health care seeking populations in five low-and middle-income countries: a cross-sectional study. Epidemiology and psychiatric sciences. 2018;27(4):393.
26. Hjelmeland H, Knizek BL. The emperor's new clothes? A critical look at the interpersonal theory of suicide. Death studies. 2019.
27. Hegerl U. Prevention of suicidal behavior. Dialogues in clinical neuroscience. 2016;18(2):183.
28. Park GR, Park E-J, Jun J, Kim N-S. Association between intimate partner violence and mental health among Korean married women. Public health. 2017;152:86-94.
29. Šedivy NZ, Podlogar T, Kerr DC, De Leo D. Community social support as a protective factor against suicide: a gender-specific ecological study of 75 regions of 23 European countries. Health & place. 2017;48:40-6.

track changes

Click here to access/download
Supporting Information
Sucide and polygamy track changes.docx
