# Supplementary material for: The prevalence of suicidal behavior and its associated factors among wives with polygamy marriage living in Gedeo zone, southern Ethiopia, 2020
Source: PLoS One. 2021 Oct 25;16(10):e0259029. doi: 10.1371/journal.pone.0259029 (PMC8544854; doi:10.1371/journal.pone.0259029)
Supplement: Attachment — Submitted filename: PLoS One_Summary of Peer reviewing.docx [file pone.0259029.s002.docx]

**Summary of Peer reviewing**

**Name of Peer Reviewer:** Monica Ewomazino Akokuwebe, PhD

**Affiliation:** North West University, Mafikeng Campus, South Africa

**Email:** [monica.akokuwebe@gmail.com](mailto:monica.akokuwebe@gmail.com)

**Phone:** +2348061381792

1. **Summary of the research** (summarizing the main research question, claims, and conclusions of the study. Also, providing the context for how this research fits within the existing literature.)

The study titled: **“The prevalence of suicidal behavior and its associated factors among wives with polygamy marriage living in Gedeo zone, Southern Ethiopia, 2020”** is a topical issue that is addressing a social concern. In the era of globalization and in the fourth industrial revolution epoch. Polygamy and suicidal behaviour is becoming increasingly common, especially in African countries. Accurate data regarding the polygamy scope in Africa is limited and its prevalence varies widely from country to country. Historically, factors such as demographic and socioeconomic transitions have been identified that may appear to perpetuate polygamy, hence suicidal behaviour follows when the marriage is no longer palatable, especially among younger women. Thus, this study has brought out its relevance by carrying out a study using Ethiopia as a case study. Ethiopia is faced with growing prevalence of polygamy marriages as well as suicide behaviour among women in polygamous marriages (I have inserted some Readings in the main work pop up comments).

**Manuscript’s strengths:** The strength of the manuscript was a study carried out as a cross-sectional survey and also one of the few studies on this topical issue above discussed.

**Manuscript’s weaknesses:** The manuscript weaknesses was the author had failed to include prevalences of the topical issues and the introduction and methodology is lagging behind (See all my comments on the pop up comment chat on the main body of the paper).

2. **Major issues**

Major issues were inserted in the body of work (see the yellow pop up comment chat).

**Minor issues**

Minor issues were inserted in the body of work (see the yellow pop up comment chat).

3. **Other points:** Get more readings from African countries on polygamy marriage and suicidal behaviour.

4. Overall recommendation: I recommend this study titled: **“The prevalence of suicidal behavior and its associated factors among wives with polygamy marriage living in Gedeo zone, Southern Ethiopia, 2020”** for publication with major revision (See all comments and suggested readings that will help the work).
