# Supplementary material for: The prevalence of suicidal behavior and its associated factors among wives with polygamy marriage living in Gedeo zone, southern Ethiopia, 2020
Source: PLoS One. 2021 Oct 25;16(10):e0259029. doi: 10.1371/journal.pone.0259029 (PMC8544854; doi:10.1371/journal.pone.0259029)
Supplement: Attachment — Submitted filename: Response to Reviewers.docx [file pone.0259029.s004.docx]

Additional Editor Comments (if provided):
Dear Authors,
Based on the advice I suggest to revise the paper by following the reviewers' comments and suggestions.
Please resubmit the revised version.
Best regards,

Reviewers' comments:

Reviewer's Responses to Questions

**Comments to the Author**

1. If the authors have adequately addressed your comments raised in a previous round of review and you feel that this manuscript is now acceptable for publication, you may indicate that here to bypass the “Comments to the Author” section, enter your conflict of interest statement in the “Confidential to Editor” section, and submit your "Accept" recommendation.

Reviewer #1: (No Response)

Reviewer #3: (No Response)

2. Is the manuscript technically sound, and do the data support the conclusions?

The manuscript must describe a technically sound piece of scientific research with data that supports the conclusions. Experiments must have been conducted rigorously, with appropriate controls, replication, and sample sizes. The conclusions must be drawn appropriately based on the data presented.

Reviewer #1: Yes

Reviewer #3: Yes

3. Has the statistical analysis been performed appropriately and rigorously?

Reviewer #1: No

Reviewer #3: Yes

4. Have the authors made all data underlying the findings in their manuscript fully available?

The [PLOS Data policy](http://www.plosone.org/static/policies.action#sharing) requires authors to make all data underlying the findings described in their manuscript fully available without restriction, with rare exception (please refer to the Data Availability Statement in the manuscript PDF file). The data should be provided as part of the manuscript or its supporting information, or deposited to a public repository. For example, in addition to summary statistics, the data points behind means, medians and variance measures should be available. If there are restrictions on publicly sharing data—e.g. participant privacy or use of data from a third party—those must be specified.

Reviewer #1: No

Reviewer #3: Yes

5. Is the manuscript presented in an intelligible fashion and written in standard English?

PLOS ONE does not copyedit accepted manuscripts, so the language in submitted articles must be clear, correct, and unambiguous. Any typographical or grammatical errors should be corrected at revision, so please note any specific errors here.

Reviewer #1: Yes

Reviewer #3: Yes

6. Review Comments to the Author

Please use the space provided to explain your answers to the questions above. You may also include additional comments for the author, including concerns about dual publication, research ethics, or publication ethics. (Please upload your review as an attachment if it exceeds 20,000 characters)

1. Reviewer #1: I am not satisfied with the responses. Statistical analysis is unreliable.
The paper lacks sufficient quality for publication, I evaluated it as a whole, but I was not convinced of it, I am not satisfied with the responses the researchers did not make the modifications with the required quality.

**Author’s response: Dear reviewer, we authors want to ask excuse for your feeling of being not satisfied with our response.**

**We tried to revise again your comments and incorporate responses.**

**Note: We authors are ready to correct each specific question you want to raise for clarification and elaboration again. We authors are also ready to submit the data if you have such significant concern.**

2. Reviewer #3: There is a problem with keeping spaces consistent throughout.

**Author’s response: Thank you dear reviewer. We accept the comment and corrected accordingly.**

The fertility rate is high in this area as compared to other parts of the country. This needs to be cited.

**Author’s response: Thank you dear reviewer.**

**The reference you asked was cited properly and written as follows**

**The zone has a population of 850, 534 and 500,000 of them were females. The fertility rate is higher in this area as compared to other parts of the country (22).**

**3**. Add few sentences on systematic random sampling technique. It seems quite arbitrary.

**Author’s response: Dear reviewer, we included few sentences and re- written as follows**

**Sampling Technique**

This study used a systematic random sampling technique. To get the sampling frame of the sample, we gave coding for each number households practicing polygamy marriage. There were 1692 households who practiced polygamy and living in the Gedeo zone. To calculate K^th^ interval, we used the formula (N/n), where N= Total number of population and n= required sample. K^th^ interval= (1692/423=4th) and include all samples by counting every 4^th^ interval.
Results
4. My confusion still remains as to why your age, husband's work, and educational level sections are so limited. Adding more categories would be helpful.

**Author’s response: Dear reviewer, our participant’s data was in to two for Age, Husband work and educational classification.**

**This is based on the finding of our data.**

5. It would be beneficial to add a brief paragraph to describe the outcome variables and other covariates (see the standard journal article format).

**Author’s response:** **Dear reviewer, it was corrected as follows**

**Study variables**

**Dependent/out-come variable**

Suicidal behaviour (Yes/No)

**Independent variables**

Socio-demographic variables: Age, educational status, employment, residence, husband work, wife’s house

Marriage and psycho-social variables: religion, and duration of marriage, number of wives at a time, distance of the two wife’s house, family size, social support, and duration of marriage.

6. Additionally, you were unable to refer to 1 since it is used as a reference category.
**Author’s response: we were rewritten on the bottom of the table as follows**

**(1, reference category, * p< 0.05, **, p<0.01), model fitness = 78%)**
7. Despite your acknowledgment in your comments, I have not been able to understand when you used the Chi-square test.

**Authors response: Dear reviewer one of the reviewers suggested to include chi-square**

8. It is of major concern that some variables, such as religion, were left out of the AOR model.

**Authors response: Dear reviewer, we included and corrected as follow**

| **Religion** | Protestant | 329(77.7% | 172 | 157 | 1 |  |
| --- | --- | --- | --- | --- | --- | --- |
|  | Orthodox | 64(15.1%) | 29 | 35 | 1.32(0.77- 2.26) |  |
|  | Muslim | 30(7.09) | 14 | 16 | 1.252(0.59- 2.64) |  |

9. And that some variables, such as depression, and domestic violence, were absent from the first table.

**Author’s response: Thank you, Dear reviewer; it was there in table 2**

Depression	Yes	237(56.0%)	132	105	17.6	.000027

No	186(43.9%)	65	120

Intimate partner violence	Yes	144(43.0%)	90	54	30.4	0.00001

No	279(65.9%)	96	183

10. This needs to be clearly specified. Additionally, you could replace the first table with chi-square results and 95%CI intervals, since it makes no sense to repeat percentages in both tables.
**Author’s response: Dear reviewer, we merged both table 1 and table 2 by including the Chi-square result**

| **Variables** | **Category variables** | | **Suicidal behavior** | | X^2^ | **Adjusted odds ratio (AOR), 95% CI** | **P-value** |
| --- | --- | --- | --- | --- | --- | --- | --- |
|  |  |  | Yes | No |  |  |  |
| **Age** | Below 30 | 135 | 94 | 41 | 3.1205 | 1.48(0.96, 2.29) | 0.43 |
|  | Above 30 | 288 | 175 | 113 |  | 1 |  |
| **Educational status** | Non formal(illiterate) | 309 | 211 | 98 | 34.7904 | 3.83 (2.44-6.02) | 0.001* |
|  | Primary school  (grade 1-8) | 114 | 41 | 73 |  | 1 |  |
| **Residence** | Urban | 42 | 19 | 23 | 3.22 | 0.53(0.28,1.01) | 0.13 |
|  | Rural | 381 | 232 | 149 |  | 1 |  |
| Religion | Protestant | 329(77.7% | 172 | 157 | 1.6788 | 1 |  |
|  | Orthodox | 64(15.1%) | 29 | 35 |  | 1.32(0.77- 2.26) |  |
|  | Muslim | 30(7.09) | 14 | 16 |  | 1.252(0.59- 2.64) |  |
| **Husbands work** | Farmer | 313 | 140 | 173 | 0.83 | 1 |  |
|  | Merchant | 110 | 43 | 67 |  | 0.79(0.51,1.24) | 0.24 |
| **Wives work** | House wife | 250 | 134 | 116 | 5.71 | 1 |  |
|  | Farmer | 89 | 39 | 50 |  | 0.68(0.41,1.10) | 0.10 |
|  | Merchant | 84 | 52 | 32 |  | 1.41(0.85, 2.33) | 0.37 |
| **Number of wives at a time** | Two | 300 | 132 | 168 | 13.4 | 1 |  |
|  | Three | 123 | 79 | 44 |  | 2.29(1.48,3.53) | 0.01* |
| **Distance of the two wives house** | < 20 km | 148 | 94 | 54 | 0.67 | 1.21(0.80,1.83) | 0.15 |
|  | >20 km | 275 | 162 | 113 |  | 1 |  |
| **Family size** | <3 | 157 | 95 | 62 | 0.56 | 1.17(0.78,1.74) | 0.17 |
|  | >3 | 266 | 151 | 115 |  | 1 |  |
| **Depression** | Yes | 237 | 132 | 105 | 17.6 | 2.37(1.60-3.52) | 0.001** |
|  | No | 186 | 65 | 120 |  | 1 |  |
| **Intimate partner violence** | Yes | 144 | 90 | 54 | 30.4 | 3.18(2.09,4.83) | 0.02* |
|  | No | 279 | 96 | 183 |  | 1 |  |
| **Social support** | Poor | 182 | 106 | 76 | 24.7 | 3.02(1.92, 4.75) | 0.01* |
|  | Moderate | 89 | 36 | 53 |  | 1.47 (0.85,2.54) | 0.29 |
|  | Strong | 152 | 48 | 104 |  | 1 |  |
| **Duration of marriage** | < 3 year | 63 | 38 | 25 | 1.27 | 0.94(0.53, 1.67) | 0.11 |
|  | 3-6 year | 148 | 99 | 49 |  | 1.25(0.80, 1.94) | 0.14 |
|  | >6year | 212 | 131 | 81 | 3.1205 | 1 |  |

Discussion
11. Why haven't you compared your results with previous studies in Africa in the Discussion?
Is that due to the lack of studies? As well, I suggest reviewing a standard discussion of a journal article that explains the results critically rather than simply reporting them.

**Author’s response: Thank you very much for great suggestions.**

**We included African studies such as South Africa, Uganda and Ghana**

The study should also be mentioned with its strengths and limitations -which is a glaring omission.

**Author’s response: Thank you very much. We accept the comment and added the strength and limitation of our study in the revised manuscript.**

7. PLOS authors have the option to publish the peer review history of their article ([what does this mean?](https://journals.plos.org/plosone/s/editorial-and-peer-review-process#loc-peer-review-history)). If published, this will include your full peer review and any attached files.
If you choose “no”, your identity will remain anonymous but your review may still be made public.
**Do you want your identity to be public for this peer review?** For information about this choice, including consent withdrawal, please see our [Privacy Policy](https://www.plos.org/privacy-policy).

Reviewer #1: **Yes:** suzan Abdel-Rahman mohamed

Reviewer #3: **Yes:** Gayathri Abeywickrama

[NOTE: If reviewer comments were submitted as an attachment file, they will be attached to this email and accessible via the submission site. Please log into your account, locate the manuscript record, and check for the action link "View Attachments". If this link does not appear, there are no attachment files.]

While revising your submission, please upload your figure files to the Preflight Analysis and Conversion Engine (PACE) digital diagnostic tool, <https://pacev2.apexcovantage.com/>. PACE helps ensure that figures meet PLOS requirements. To use PACE, you must first register as a user. Registration is free. Then, login and navigate to the UPLOAD tab, where you will find detailed instructions on how to use the tool. If you encounter any issues or have any questions when using PACE, please email PLOS at [figures@plos.org](mailto:figures@plos.org). Please note that Supporting Information files do not need this step.
